# Supplementary material for: Nitrogen Removal from Landfill Leachate Using Biochar Derived from Wheat Straw
Source: Materials (Basel). 2024 Feb 17;17(4):928. doi: 10.3390/ma17040928 (PMC10890371; doi:10.3390/ma17040928)
Supplement: Supplementary file 1 [file materials-17-00928-s001.zip › materials-2857020-supplementary.pdf]

SUPPLEMENTARY

Nitrogen Removal from Landfill Leachate Using Biochar Derived from Wheat Straw

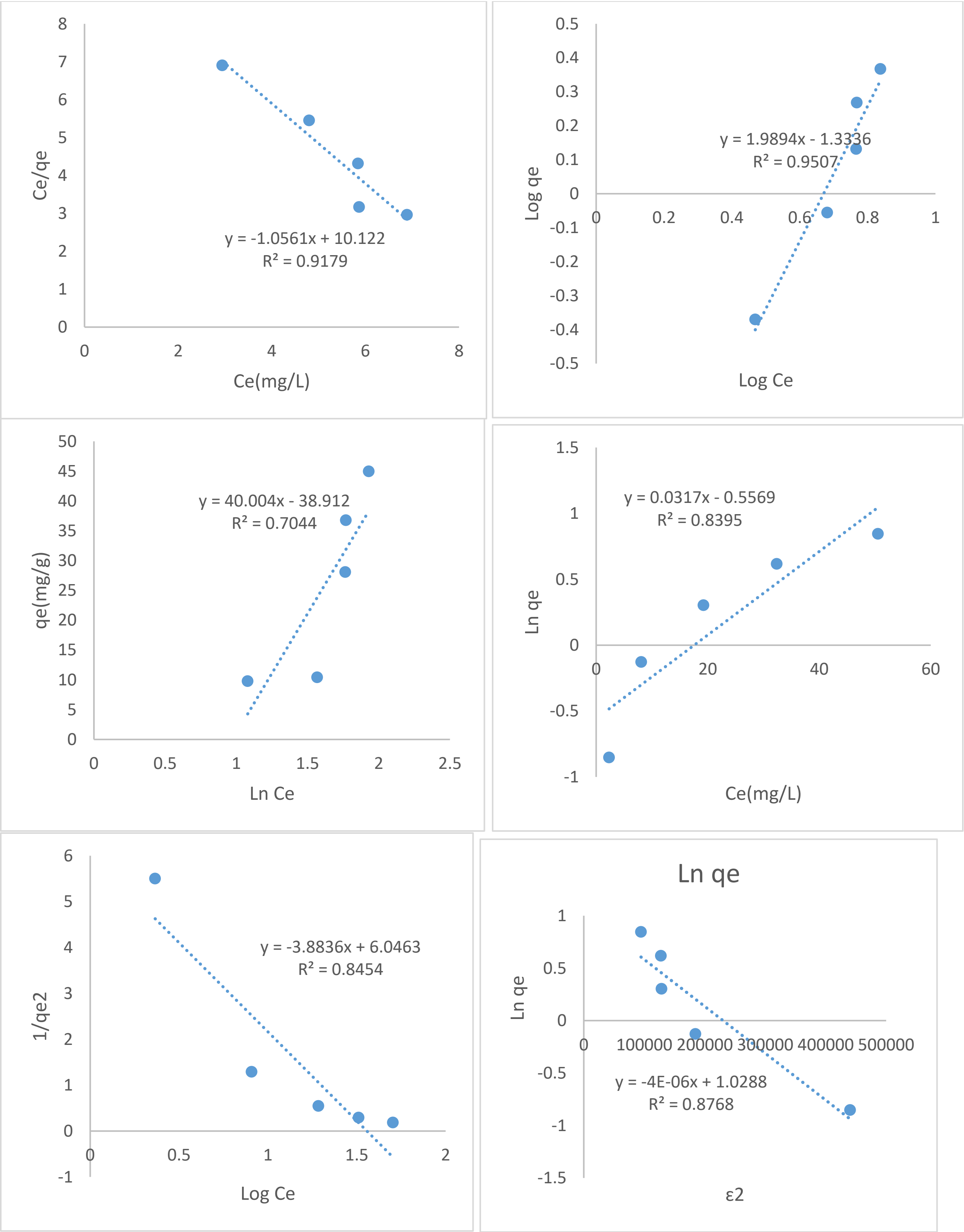

Figure S1. (a) Langmuir, (b) Freundlich, (c) Tempkin, (d) Jovanovich, (e) Harkin-Jura and (f) Dubinin-Radushkevich isotherms of nitrogen removal from NH_4Cl solution by AC.

SUPPLEMENTARY
Nitrogen Removal from Landfill Leachate Using Biochar Derived from Wheat Straw

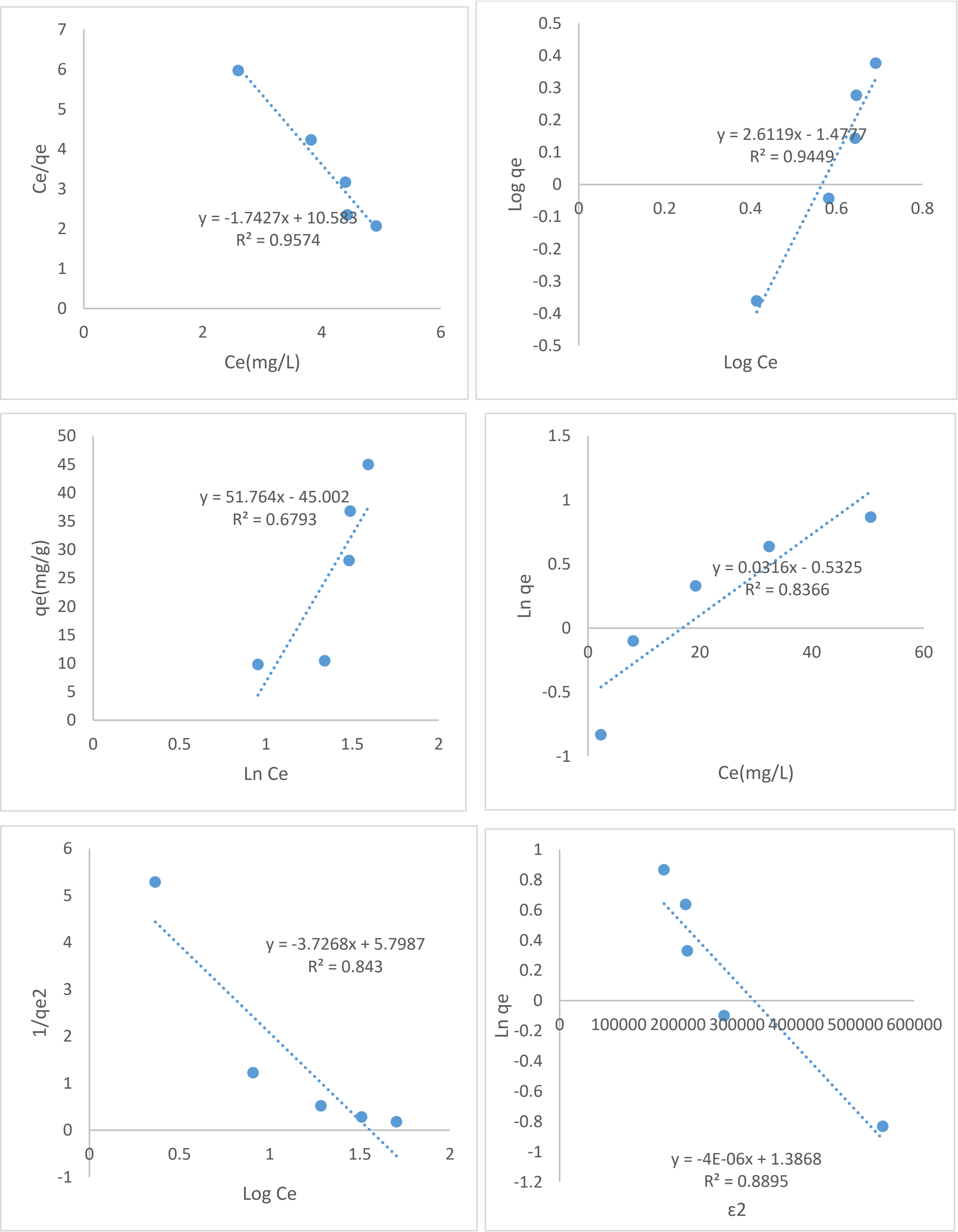

Figure S2. Langmuir, (b) Freundlich, (c) Tempkin, (d) Jovanovich, (e) Harkin-Jura and (f) Dubinin-Radushkevich isotherms of nitrogen removal from NH_4Cl solution by BC.

SUPPLEMENTARY
Nitrogen Removal from Landfill Leachate Using Biochar Derived from Wheat Straw

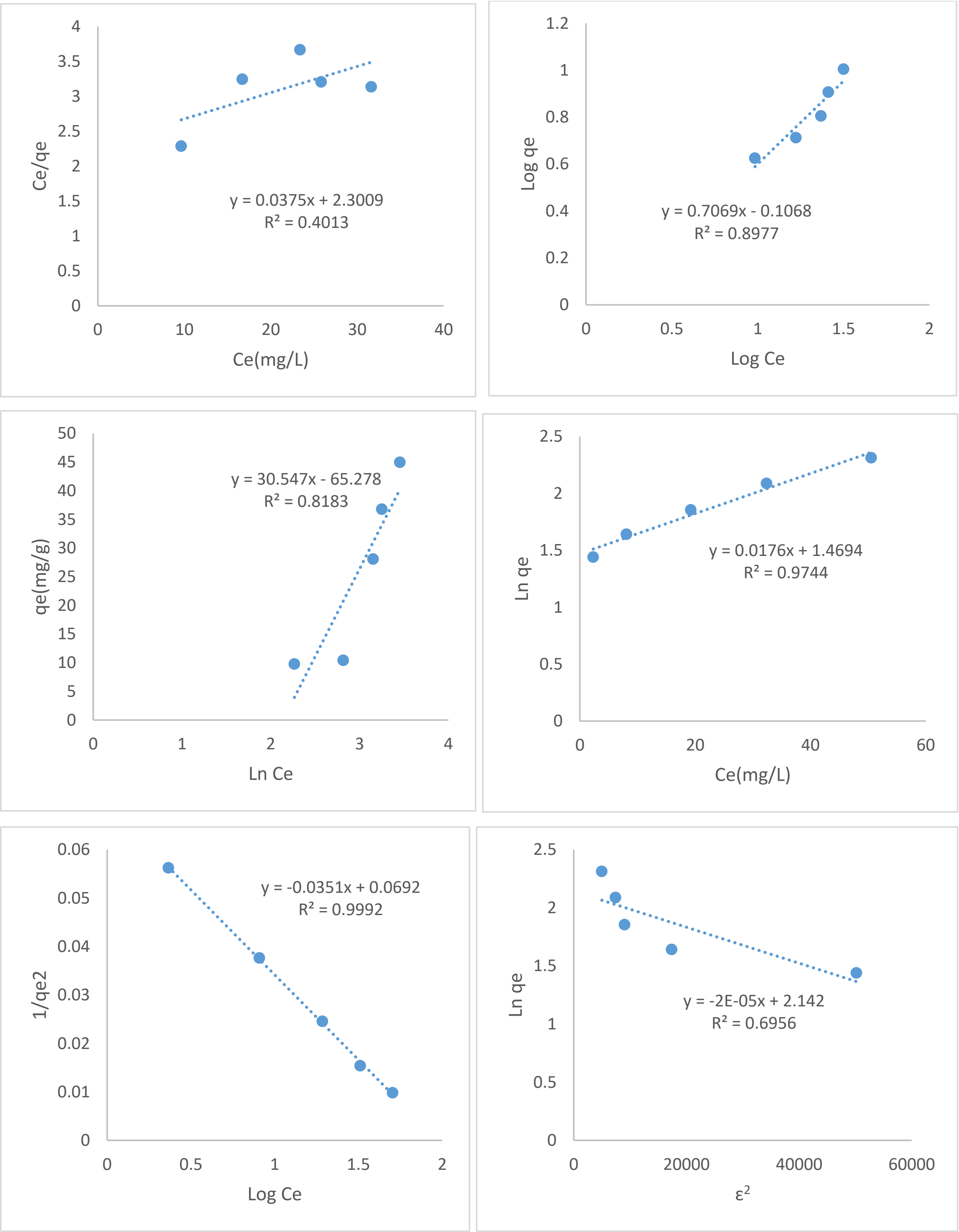

Figure S3. Langmuir, (b) Freundlich, (c) Tempkin, (d) Jovanovich, (e) Harkin-Jura and (f) Dubinin-Radushkevich isotherms of nitrogen removal from LLCH by BC.
